# Quantification of ecological complexity and resilience from multivariate biological metrics datasets using singular value decomposition entropy

**DOI:** 10.1016/j.mex.2019.07.020

**Published:** 2019-07-19

**Authors:** Antoni Ginebreda, Laia Sabater-Liesa, Damià Barceló

**Affiliations:** a Department of Environmental Chemistry, Institute of Environmental Assessment and Water Research (IDAEA-CSIC), Jordi Girona 18-26, 08034, Barcelona, Spain; b Catalan Institute for Water Research (ICRA). Carrer Emili Grahit 101, 17003, Girona, Spain

**Keywords:** Singular value decomposition entropy, Ecological resilience, Ecological complexity, Singular value decomposition, Singular value entropy, River phytoplankton, Ebro River

## Abstract

The concept of resilience has become popular in many disciplines far beyond its original use in the field of ecology. Despite of its wide use, it has received different definitions not always coincident. Such ambiguity is still more evident in its quantitative characterization. Most of the available methods are heavily context dependent and often difficult to apply in the practice. Here, we propose to define and calculate resilience starting from the data matrices resulting from multivariate measurements of different biological metrics.

• The resilience between two field scenarios (each one characterized by their corresponding datasets) can be conveniently captured as the difference between its respective data complexities.
• Complexity is quantified by means of the entropy associated to the spectral distribution of the singular values of each data matrix.
• The method proposed has been illustrated with a case study in which the resilience of a river (Ebro River, NE Spain) is calculated comparing six biological metrics associated to the phytoplankton, upstream and downstream to a series of large reservoirs that alter the natural river flow regime.

## Graphical abstract

**Figure:**
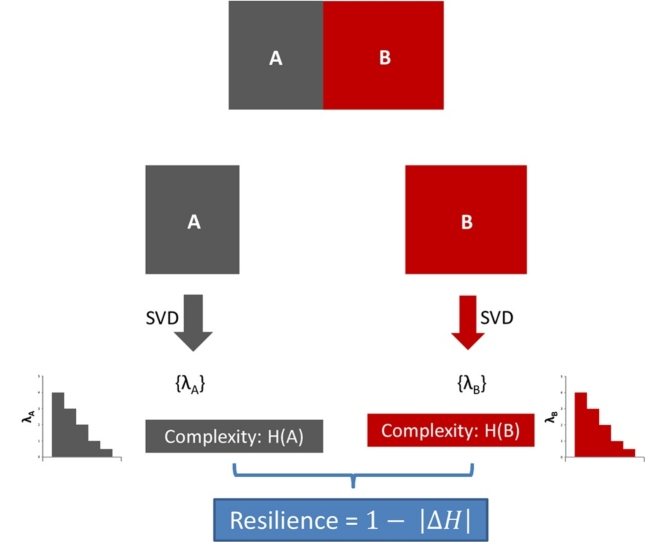

**Specifications Table**

**Table:** 

Subject Area:	Environmental Science
More specific subject area:	Freshwater Ecology
Method name:	Singular Value Decomposition Entropy
Name and reference of original method:	Singular Value Decomposition Entropy
Resource availability:	The method has been evaluated using experimental data from Sabater-Liesa et al., 2019 [20]

## Method details

The term ‘resilience’, appeared for the first time in the ecological science in 1973 [1], rapidly influenced other scientific domains such as engineering, economics, medicine or social sciences. Since that time many alternative definitions of resilience have been proposed [[2], [3], [4], [5], [6], [7]]. For the purposes of this article, we will follow Holling’s seminal concept [1], which refers to the capacity of an ecosystem to cope with changing external conditions without losing its structural and functional characteristics. Despite its broad use, the definitions and interpretations of resilience are still the matter of deep discussion in the literature [[2], [3], [4], [5], [6], [7]], particularly when they need to be applied to specific case studies. Such difficulties are particularly challenging when resilience has to be quantified. Although there are many methods reported in the literature, in general, they all tend to be strongly context-dependent so that their application is only feasible for specific experiments or scenarios [2,3]. Therefore, there is a need for general methods of resilience quantification capable of broad application and suitable to be used in the common practice of field ecology.

Data gathered from environmental biological field monitoring typically consists of measurements of different variables spanning on space and time, that are conveniently organized in the form of data matrices. Extracting information from such data matrices is a problem usually addressed from multivariate statistics [8]. Among the plethora of techniques available, here we specifically focus on the singular value decomposition (SVD) technique (see details below), which is underlying in many of the existing methods broadly used in multivariate data analysis. Furthermore, SVD has been successfully applied in a large variety of scientific and technical domains ranging from signal and image processing, genomic analysis, weather forecast, chemometrics, disease surveillance or big-data analysis [[9], [10], [11], [12], [13], [14], [15]].

**Fig. 1:**
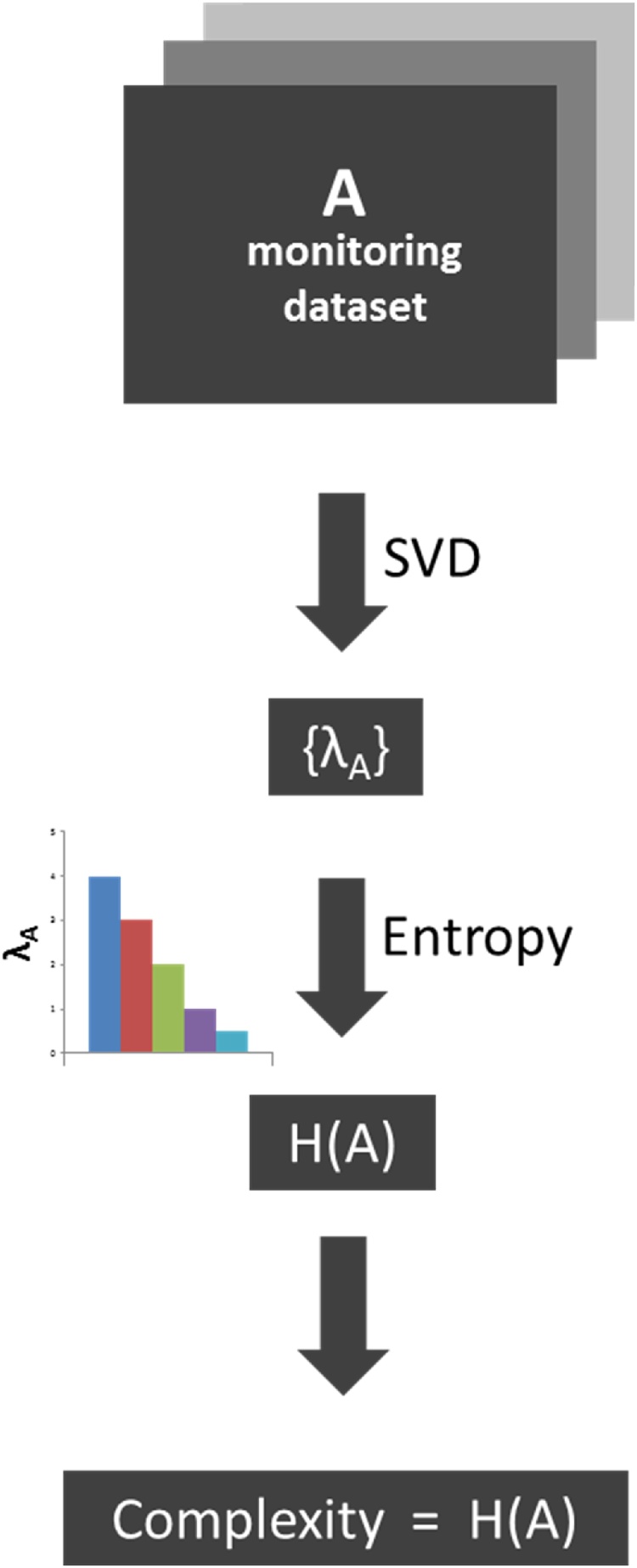
Flow-chart overview of the method showing the connection between a dataset of measurements with its complexity quantification in terms of SVD entropy. The workflow includes the following steps: (a) SVD decomposition of the monitoring dataset matrix *A* which allows obtaining the set of singular values {λ_A_} (Eqs. (1) and (2)); (b) Calculation of the SVD entropy *H(A)* (Eq. (4)) that is assimilated to the dataset complexity.

Here we are specifically interested in the characterization of the data complexity (organized in appropriate matrices of empirical measurements or derived metrics) which is assumed to quantitatively reflect the system’s own complexity. In turn, the extent of changes in system complexity between two situations or scenarios of a given system is proposed as a general quantitative empirical metric of resilience [16]. To that end, we make use of the so-called SVD entropy, which captures how is the distribution of the singular values (SVs) of the data matrix analyzed (see Fig. 1). SVD entropy has found applications in a variety of areas like econometrics [[9], [10], [11]], genome expression data processing [12], image processing [13] or medical sciences [14,15].

### Singular Value Decomposition of a data matrix (SVD)

Briefly, this technique consists of the decomposition of any *A (m × n; m ≥ n)* matrix into a product of three matrices as:

(1) $$A=U∙\text{Σ}∙V^{T}$$

where *U* is an *(m × m)*unitary matrix, *Σ* is an *(m × n)* rectangular diagonal matrix with non-negative real numbers on the diagonal, and *V* is an *(n × n)* real or complex unitary matrix and *V^T^* denotes its transpose. The diagonal entries *λ_i_* of Σ are known as the singular values of *A*. The columns of *U* and the columns of *V* are respectively called the left-singular vectors and right-singular vectors of *A*.

### SVD entropy

Following [10] it is possible to define a complexity measure of the dataset contained in matrix *A*, using the set of singular values (*λ_i_*)*_i=1,n_* by means of a suitable ‘Shannon type entropy’ [17] (Fig. 1). To do so, we first arrange the singular values (*λ_i_*)*_i=1,n_* in decreasing order and normalize them so that:

(2) $${\bar{\lambda }}_{i}=\frac{\lambda_{i}}{\sum_{i}\lambda_{i}}$$

(3) $$\text{w}\text{i}\text{t}\text{h}\;{\text{Σ}}_{i}{\bar{\lambda }}_{i}=1$$

The SVD Entropy of *A* denoted as *H(A)* is thus defined as:

(4) $$H\left(A\right)=\;-\sum_{i=1}^{n}{\bar{\lambda }}_{i}∙\text{ln}\left({\bar{\lambda }}_{i}\right)\;$$

For comparison purposes between matrices having different dimensions, *H(A)* is conveniently normalized dividing by the factor ln*(n)* which corresponds to the maximum value attainable by *H(A)*. In this way, *H(A)* is bounded between 0 and 1:

(5) $$H\left(A\right)=\;\frac{-1}{\text{ln}\left(n\right)}\sum_{i=1}^{n}{\bar{\lambda }}_{i}∙\text{ln}\left({\bar{\lambda }}_{i}\right)$$

**Fig. 2:**
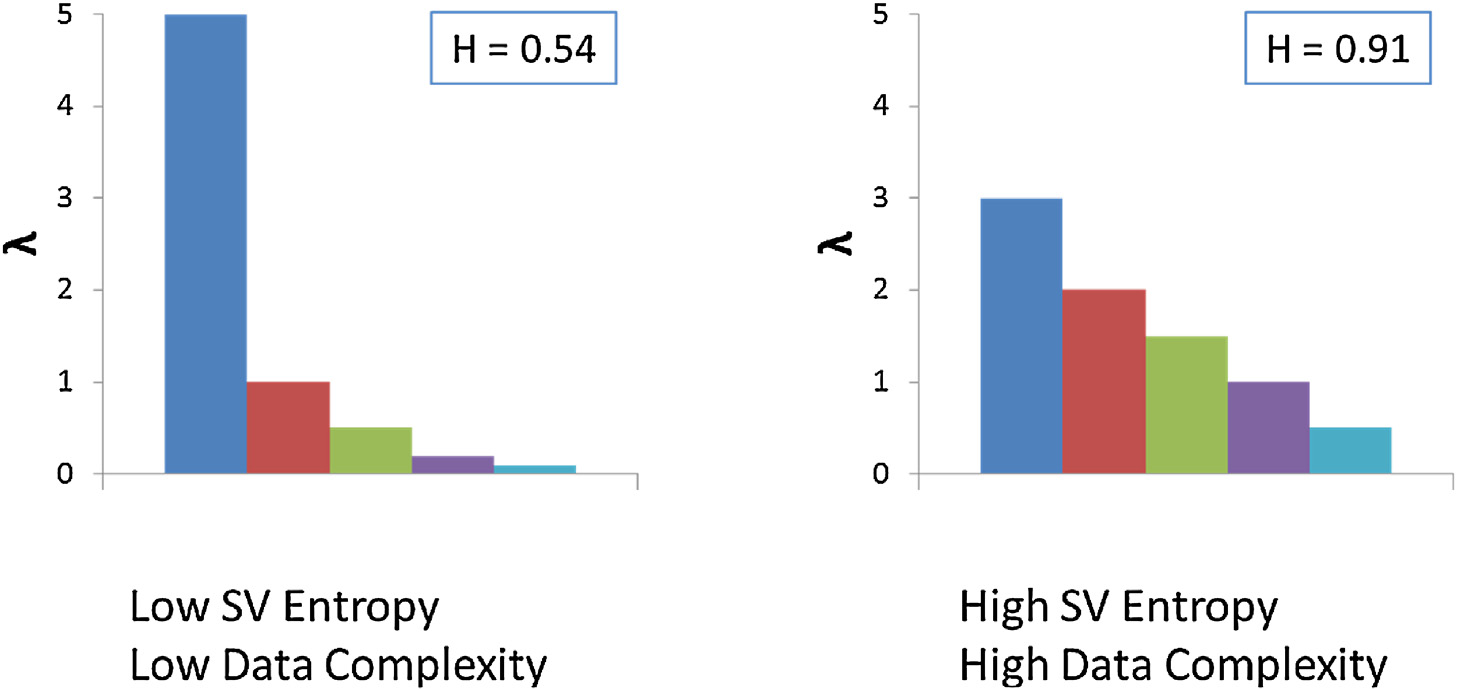
Two hypothetical distributions of singular values (arbitrary scale) highlighting a low and high entropy (complexity) profiles and their respective entropies (calculated using Eq. (5)).

Fig. 2 shows two hypothetical examples of singular values distribution with their respective entropies calculated using Eq. (5).

### SVD entropy and resilience

For a given variable, the resilience quantification proposed here involves comparing two related scenarios, each one characterized by its corresponding data matrix, using SVD entropy in terms of increase/decrease of the dataset complexity. i.e., a lower entropy reflects a non-uniform distribution of the singular values *λ_i_* thus corresponding to low-complexity of the underlying data; conversely, higher SVD entropy denotes that the set of *λ_i_* is more evenly distributed (Fig. 2).

A system that is able to maintain its complexity after a perturbation will be qualified as ‘resilient’, while the opposite behavior would be indicative of a lack of resilience. Let us considered a system in two states A and B, each one characterized by the corresponding matrices of measurements or metrics of their respective variables. The difference in complexity between states A and B of such a system (expressed as the corresponding difference on SVD entropies) can be related to the system’s resilience. Since high resilience is associated with low changes in data complexity, a suitable and general measure of resilience can be conveniently captured by the following equation:

(6) $$Resilience\;\left(A,B\right)=1-\left|H\left(A\right)-H\left(B\right)\right|=1-\left|\Delta H\right|$$

**Fig. 3:**
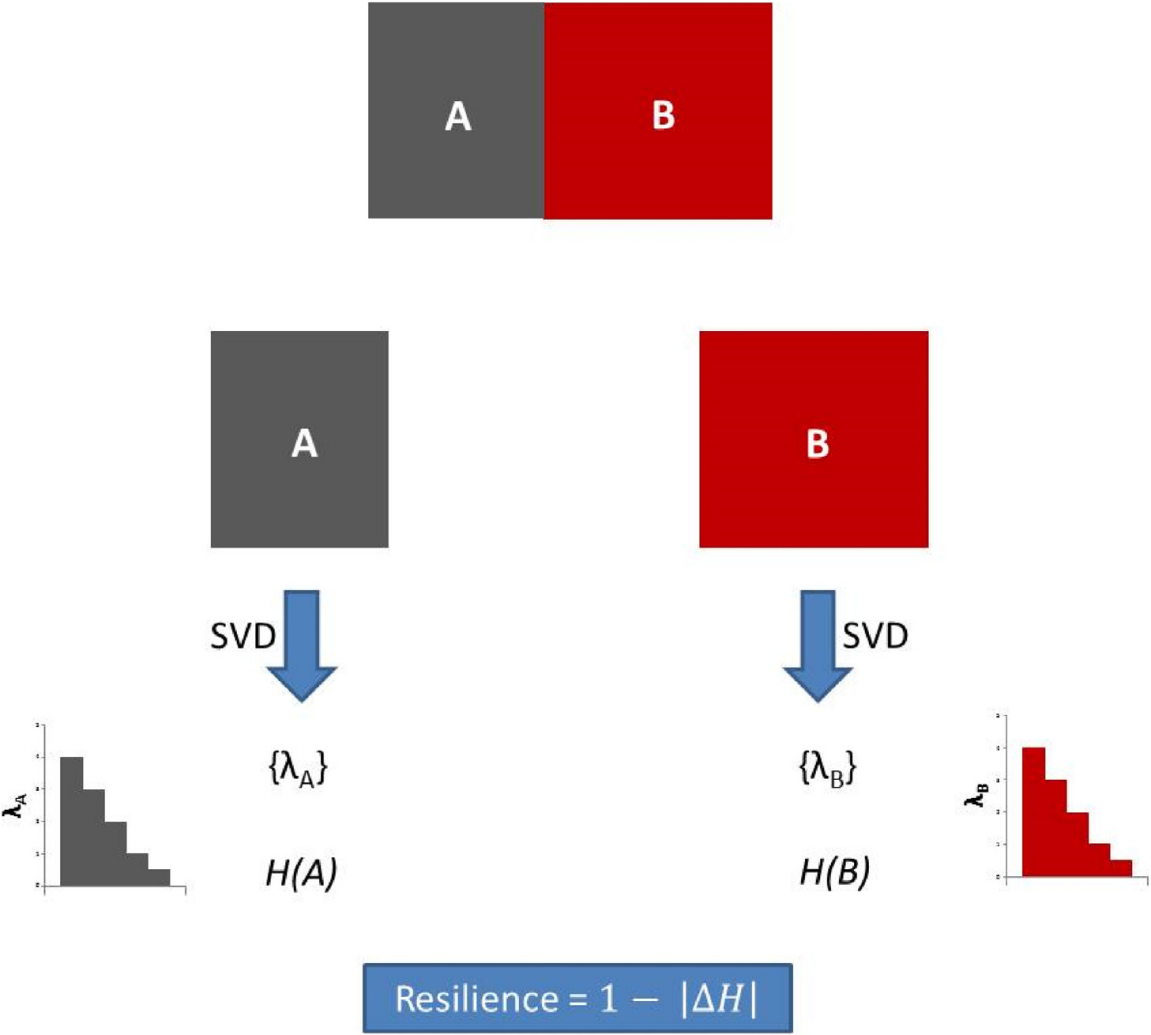
Flowchart for the calculation of resilience, comparing the variation of entropy (complexity) between two related data matrices. The workflow includes the following steps: (a) SVD decomposition of the two monitoring dataset matrices *A* and *B*, which allows obtaining the sets of singular values {λ_A_} and {λ_B_} (Eqs. (1) and (2)); (b) Calculation of the SVD entropies *H(A)* and *H(B)* (Eq. (4)) that are assimilated to the respective datasets complexities; (c) Calculation of the system’s resilience (Eq. (6)).

Since *H* is always comprised between 0 and 1, this resilience index is comprised between 0 and 1 too. Resilience equals 1 if *H(A) = H(B)* corresponding to a lack of change in complexity between A and B scenarios, and thus to a maximum resilience. Conversely, if *H(A) = 1* and *H(B) = 0* (or the opposite) then resilience becomes 0 thus reflecting a maximum change in complexity between the scenarios compared. The whole process is summarized in Fig. 3.

### Method validation using a case study

The foregoing method was tested in a stretch of the Ebro River basin (NE Spain). The Ebro basin is located in the Northeastern part of the Iberian Peninsula occupying a total surface of 85362 km^2^. The main river is 910 km length and flows from the Cantabrian Mountains to the Mediterranean Sea. In terms of water flow the Ebro River is the largest one in the Iberian Peninsula (mean annual discharge 435 m^3^ s^−1^). The middle course of Ebro mainstream is affected by three consecutive large reservoirs, Mequinenza (1500 Hm^3^), Riba-roja (210 Hm^3^) and Flix (11 Hm^3^) [18,19], causing major changes in the hydromorphological dynamics (flood peaks alteration, retention of sediments, etc.) that are reflected on the ecological status of the river. The purpose of our exercise aimed at quantifying the system resilience comparing the data measured upstream and downstream to the reservoirs.

**Fig. 4:**
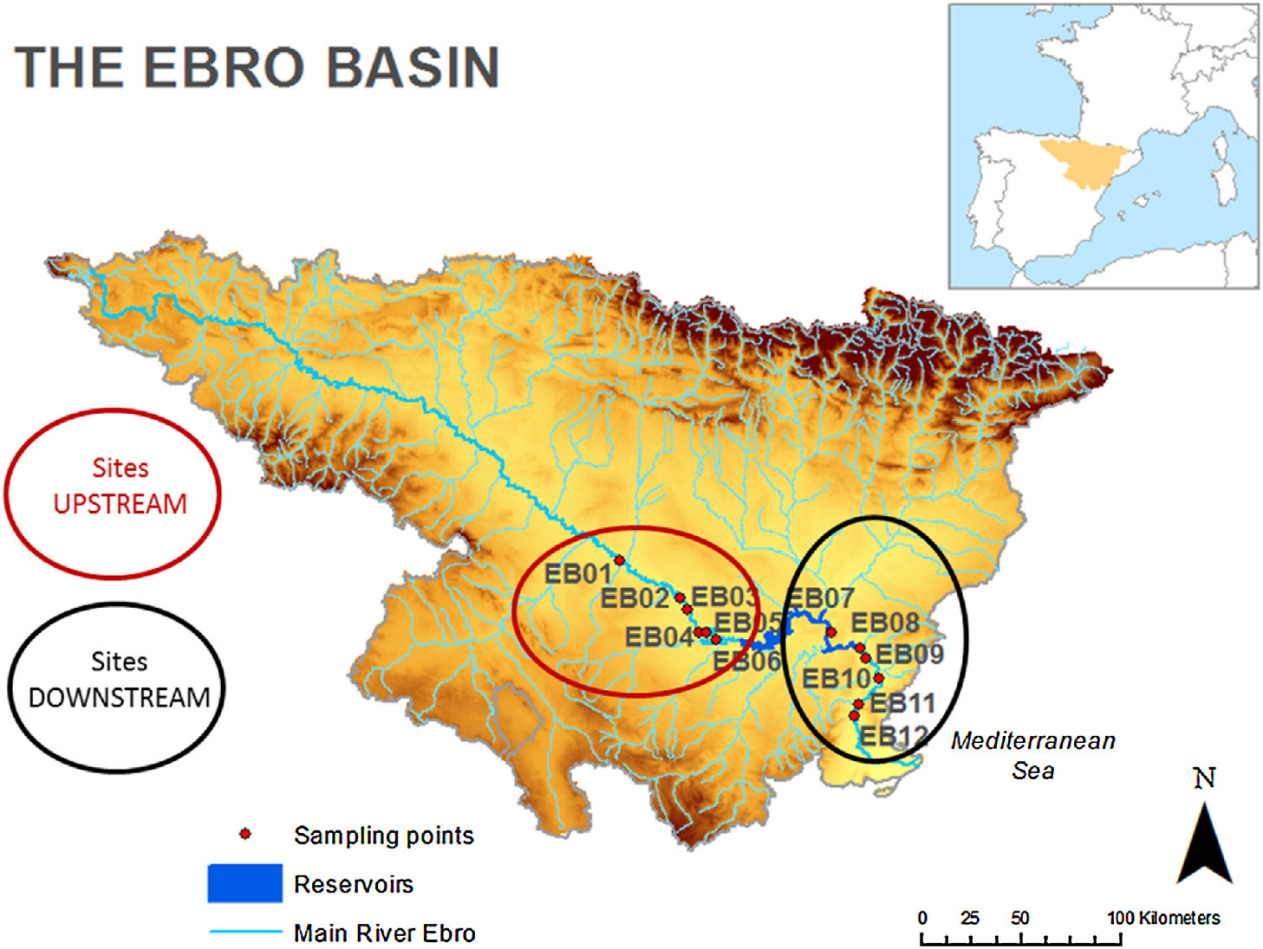
Area of study: The Ebro river middle course, showing the sampling points located upstream and downstream to the reservoirs.

Biological data used in the present study were published elsewhere [[20], [21], [22], [23]]. Twelve sites located in the mid-lower course from Zaragoza to the proximity of the river mouth were selected (Fig. 4). The first six sites were located upstream to the reservoirs, while the remaining were downstream. Six biological variables related to the phytoplankton were considered. They included metrics related to the algal community structure (Shannon-Wiener diversity, number of species, cell density, biovolume, and chlorophyll-*a* concentration) and function (alkaline phosphatase activity, APA). Datasets used can be found in [23].

**Fig. 5:**
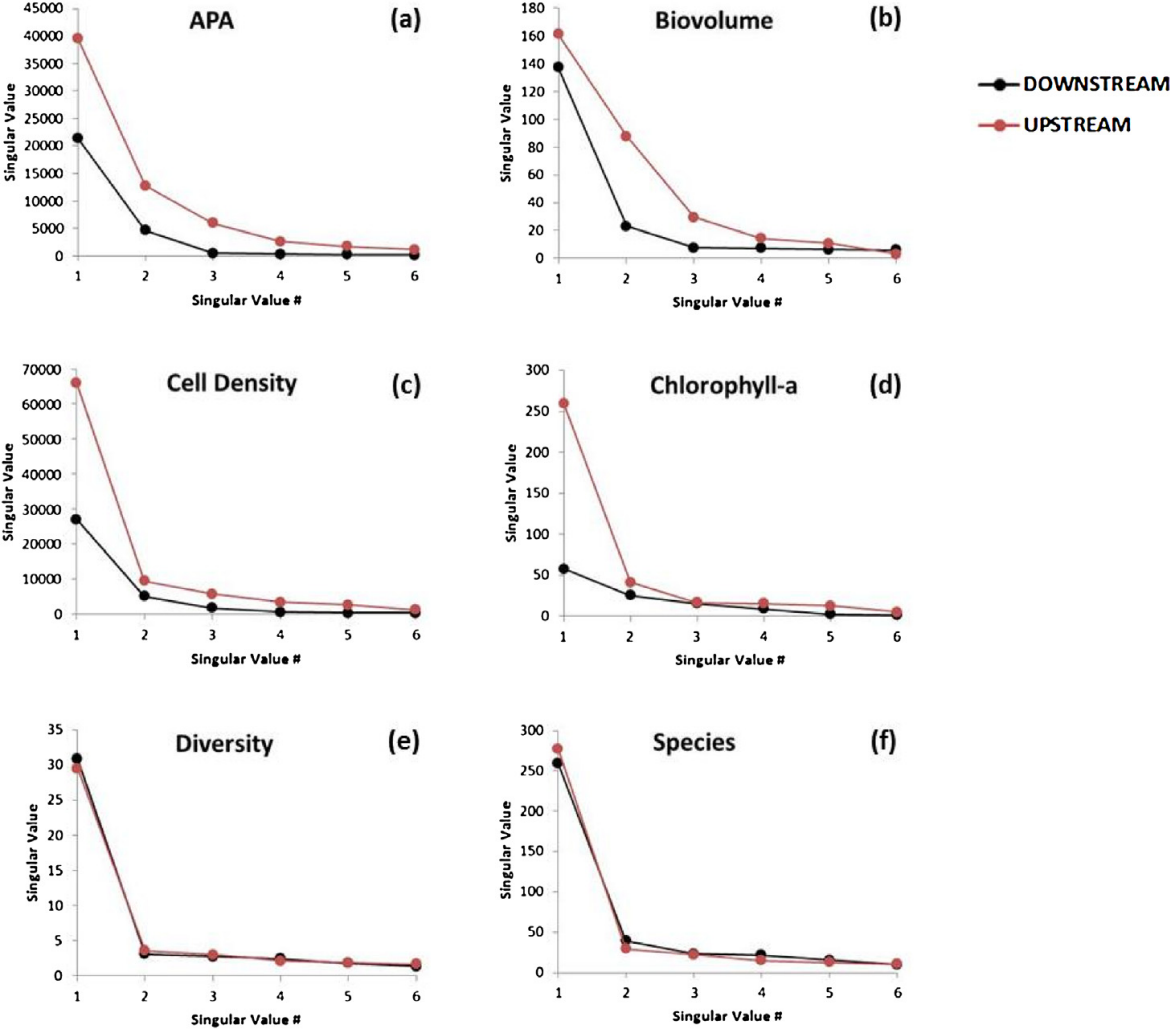
Distribution of the singular values for the biological metrics considered, upstream and downstream to the reservoirs. Note that the scales of the vertical axis are different for each biological metrics.

**Fig. 6:**
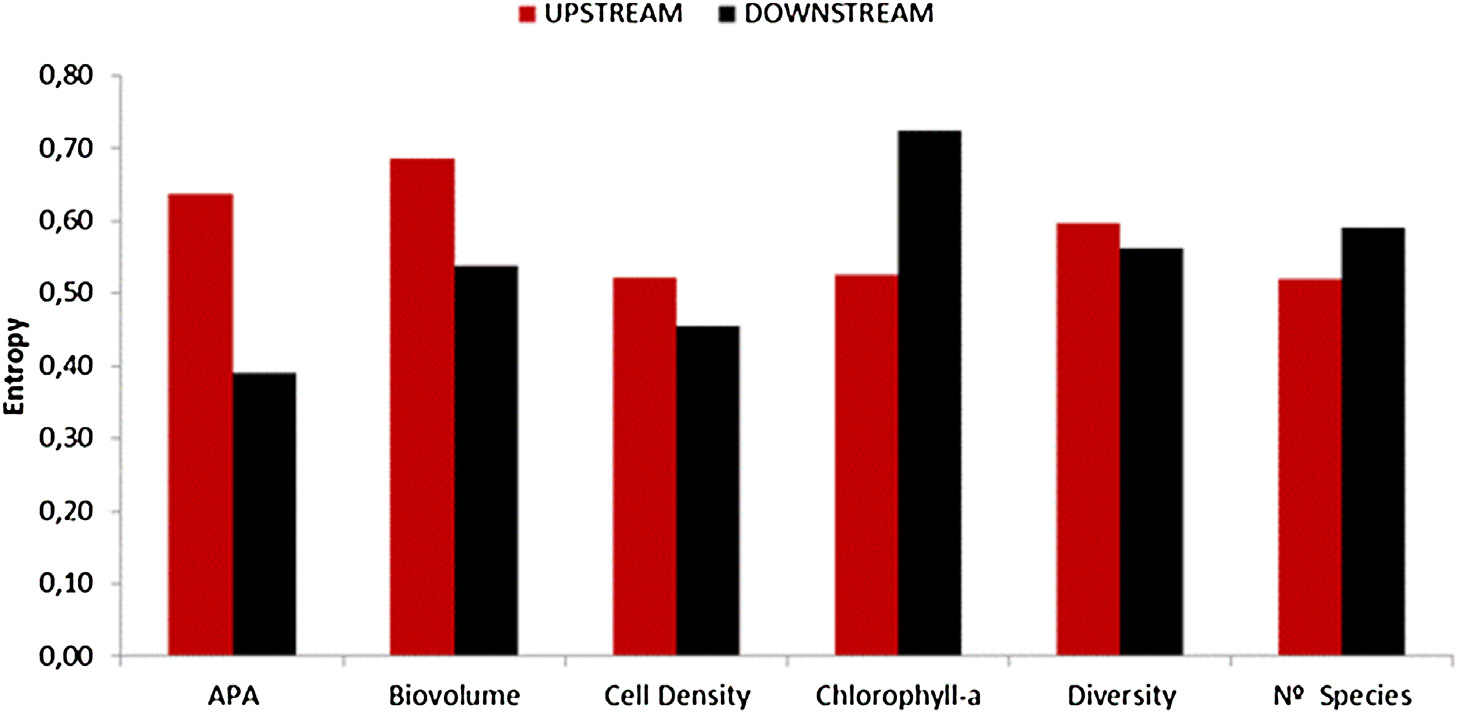
Comparison of entropy (complexity) values between upstream and downstream sites for the different biological metrics studied.

We constructed two dataset matrices for every measured biological variable, for the upstream and downstream sites respectively. Every matrix is constituted by a table of *sites × time*. These are handled as rectangular matrices of *m* columns (*m*: number of spatial sites) and *n* rows (*n*: number of campaigns). The method outlined above was applied to each of the six metrics considered. The main results are summarized in Table 1 and Fig. 5, Fig. 6.

**Table 1:** Results of complexity (singular value entropy), resilience (complexity maintenance), for the biological metrics considered upstream (UP) and downstream (DOWN) to the reservoirs.

METRICS	SVD-Entropy (Complexity)		Resilience
	H (UP)	H (DOWN)	1 − |ΔH|
APA	0.638	0.389	0.751
Biovolume	0.685	0.538	0.853
Cell Density	0.521	0.457	0.936
Chlorophyll-a	0.525	0.723	0.802
Diversity	0.596	0.562	0.967
Number of Species	0.520	0.590	0.930

The spectra or distribution of the singular values used in the calculation of the entropies of the biological metrics considered is shown in Fig. 5. The entropies, calculated using Eq. (5), had values in the range 0.38–0.72 (i.e., 38% to 72% of its maximum value). Four out of the six variables measured (all except chlorophyll-a and, in a less extent, the number of species) exhibited higher entropy (complexity) in the sites located upstream to the reservoirs (and thus subjected to a more natural hydrologic regime) than those located downstream (regulated regime) (Table 1, Fig. 5). Resilience was quantified using Eq. (6) for the six biological metrics studied. Values obtained were in the range 0.75–0.97, that correspond to APA and diversity respectively. The high resilience values obtained for diversity (0.967) and the number of species (0.930) is also perceptible from the tight closeness of the singular values distribution for the upstream and downstream as shown in Fig. 5e and f. Altogether, the medium to high resilience values quantified indicates that the system is likely capable to recover its complexity after the perturbation caused by the reservoirs, at least for the six variables examined. A deeper discussion and interpretation of the results can be found in [23].

In summary, the foregoing example highlights the generality and broad applicability of the proposed method of resilience quantification consisting of comparing the complexity of two data blocks (matrices) in terms of their respective singular value entropy.
